# Supplementary material for: Unlocking Andean sigmodontine diversity: five new species of Chilomys (Rodentia: Cricetidae) from the montane forests of Ecuador
Source: PeerJ. 2022 Apr 19;10:e13211. doi: 10.7717/peerj.13211 (PMC9029390; doi:10.7717/peerj.13211)
Supplement: Supplemental Information 1 [file peerj-10-13211-s001.docx]

Supplementary S1. DNA Extraction Protocols.

**Cytb**

- Bonvicino & Moreira (2001)

MVZ05: CGAAGCTTGATATGAAAAACCATCGTTG

MVZ16H: AAATAGGAARTATCAYTCTGG

1 ciclo: 94°C por 2 min
35 ciclos: 93°C por 2 min, 45°C por 1.5 min, 72°C por 2 min
1 ciclo: 72°C por 5 min

- Smith & Patton (1999)

MVZ05: CGAAGCTTGATATGAAAAACCATCGTTG

MVZ14: GGTCTTCATCTYHGGYTTACAAGAC

1 ciclo: 94°C por 5 min
32 ciclos: 93°C por 15 seg, 45°C por 1.5 min, 72°C por 2 min
1 ciclo: 72°C por 10 min

**COI (Cocktail)**

- Ivanova et al. (2007)

LepF1: TGTAAAACGACGGCCAGTATTCAACCAATCATAAAGATATTGG

VF1: TTCTCAACCAACCACAAAGACATTGG

VF1d: TTCTCAACCAACCACAARGAYATYGG

VF1i: TTCTCAACCAACCAIAAIGAIATIGG

LepR1_t1: CAGGAAACAGCTATGACTAAACTTCTGGATGTCCAAAAAATCA

VR1: TAGACTTCTGGGTGGCCAAAGAATCA

VR1d: TAGACTTCTGGGTGGCCRAARAAYCA

VR1i: TAGACTTCTGGGTGICCIAAIAAICA

1 ciclo: 94°C por 1 min

5 ciclos: 94°C por 30 seg, 45°C por 40 seg, 72°C por 1 min

35 ciclos: 94°C por 30 seg, 51°C por 40 seg, 72°C por 1 min

1 ciclo: 72°C por 10 min
