# Supplementary figures and images for: Unlocking Andean sigmodontine diversity: five new species of Chilomys (Rodentia: Cricetidae) from the montane forests of Ecuador
Source: PeerJ. 2022 Apr 19;10:e13211. doi: 10.7717/peerj.13211 (PMC9029390; doi:10.7717/peerj.13211)

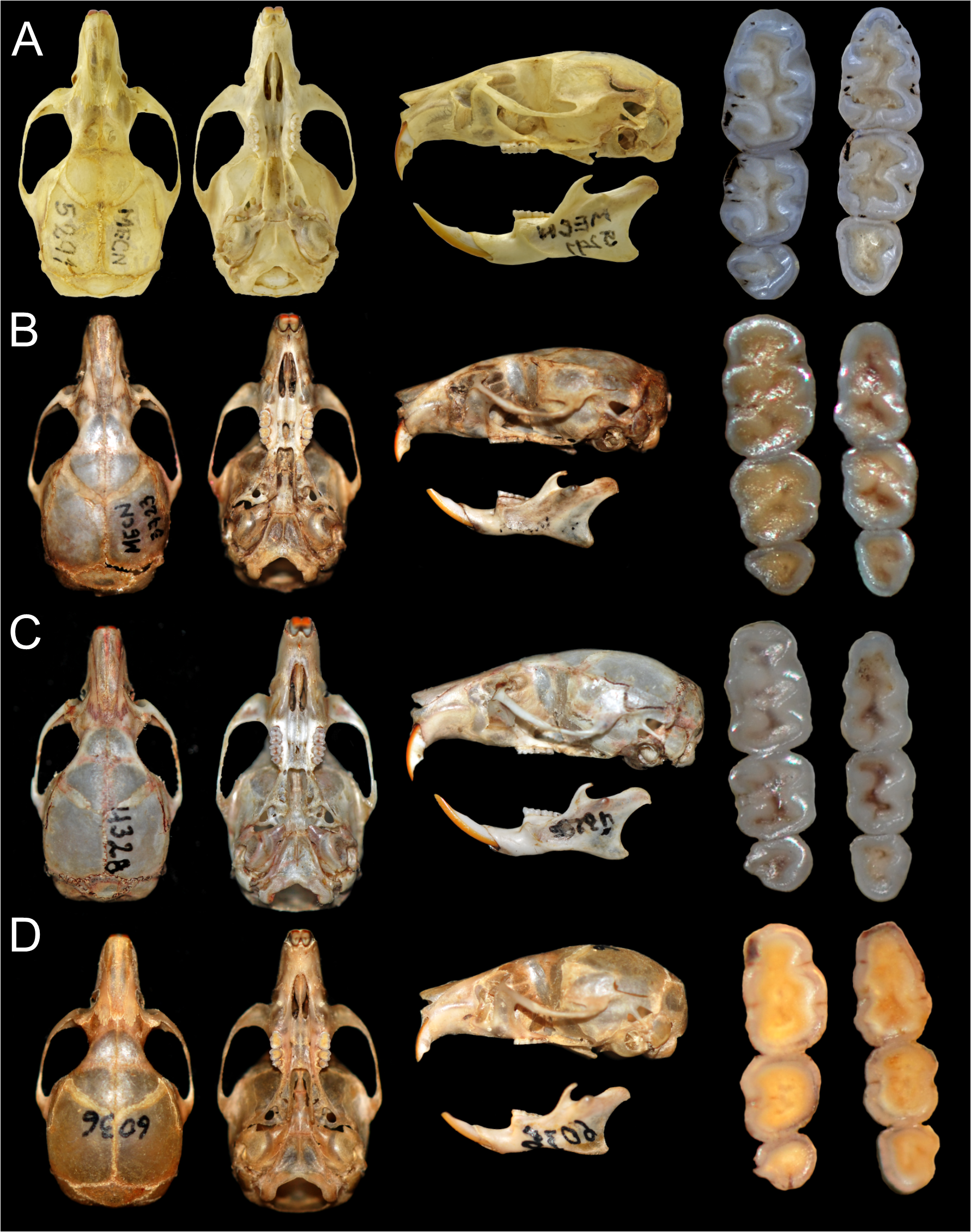

Supplement: Supplemental Information 3 — (A) C. carapazi sp. nov. (MECN 5291, holotype); (B) C. neisi sp. nov. (MECN 3723, paratype); (C) C. percequilloi sp. nov. (MECN 4328, paratype); (D) C. weksleri sp. nov. (MEPN 6036, paratype). [file peerj-10-13211-s003.png]
