# Supplementary material for: Unlocking Andean sigmodontine diversity: five new species of Chilomys (Rodentia: Cricetidae) from the montane forests of Ecuador
Source: PeerJ. 2022 Apr 19;10:e13211. doi: 10.7717/peerj.13211 (PMC9029390; doi:10.7717/peerj.13211)
Supplement: Supplemental Information 4 [file peerj-10-13211-s004.docx]

**Supplementary S4:** Stomach content composition of *Chilomys*.

| **Museum number** | **Species** | **Stomach contents** | **Total** |
| --- | --- | --- | --- |
| MECN 6315, 6205, 6181, 6303, 6327, 6323 | *C. georgedecii* | **INSECTA** |  |
|  |  | Diptera (Larvae) | 7 (50%) |
|  |  | Coleoptera (Larvae) | 4 (28.5%) |
|  |  | Hymenoptera (Larvae) | 1 (7.1%) |
|  |  | Blattodea (Larvae) | 1 (7.1%) |
|  |  | **ANNELIDA** | 1 (7.1%) |
|  |  | Undetermined arthropods |  |
|  |  | Undetermined vegetation |  |
|  |  | Total | **14 (100 %)** |
| MECN 6362, 6361, 6338 | *C. percequilloi* | **INSECTA** |  |
|  |  | Lepidoptera (Larvae) | 1 (25%) |
|  |  | Blattodea (Larvae) | 1 (25%) |
|  |  | Diptera (Larvae) | 1 (25%) |
|  |  | **ARACHNIDA** |  |
|  |  | Acari | 1 (25%) |
|  |  | Undetermined vegetation |  |
|  |  | Undetermined arthropods |  |
|  |  | Total | **4 (100%)** |
| MECN 6187 | *C. teskai* | **INSECTA** |  |
|  |  | Coleoptera (Larvae) | 1 (50%) |
|  |  | Chrysomelidae (Adult) | 1 (50%) |
|  |  | **ANNELIDA** (parasito) |  |
|  |  | Undetermined vegetation |  |
|  |  | Undetermined arthropods |  |
|  |  | Total | **2 (100%)** |
